# Noradrenergic Dysfunction in Alzheimer's and Parkinson's Diseases—An Overview of Imaging Studies

**DOI:** 10.3389/fnagi.2018.00127

**Published:** 2018-05-01

**Authors:** Andrew C. Peterson, Chiang-shan R. Li

**Affiliations:** ^1^Frank H. Netter MD School of Medicine, Quinnipiac University, North Haven, CT, United States; ^2^Department of Psychiatry, Yale University School of Medicine, New Haven, CT, United States; ^3^Department of Neuroscience, Yale University School of Medicine, New Haven, CT, United States; ^4^Interdepartmental Neuroscience Program, Yale University School of Medicine, New Haven, CT, United States

**Keywords:** norepinephrine, dopamine, neurodegeneration, neurodegenerative, locus coeruleus, ventral tegmental area, midbrain, MRI

## Abstract

Noradrenergic dysfunction contributes to cognitive impairment in Alzheimer's Disease (AD) and Parkinson's Disease (PD). Conventional therapeutic strategies seek to enhance cholinergic and dopaminergic neurotransmission in AD and PD, respectively, and few studies have examined noradrenergic dysfunction as a target for medication development. We review the literature of noradrenergic dysfunction in AD and PD with a focus on human imaging studies that implicate the locus coeruleus (LC) circuit. The LC sends noradrenergic projections diffusely throughout the cerebral cortex and plays a critical role in attention, learning, working memory, and cognitive control. The LC undergoes considerable degeneration in both AD and PD. Advances in magnetic resonance imaging have facilitated greater understanding of how structural and functional alteration of the LC may contribute to cognitive decline in AD and PD. We discuss the potential roles of the noradrenergic system in the pathogenesis of AD and PD with an emphasis on postmortem anatomical studies, structural MRI studies, and functional MRI studies, where we highlight changes in LC connectivity with the default mode network (DMN). LC degeneration may accompany deficient capacity in suppressing DMN activity and increasing saliency and task control network activities to meet behavioral challenges. We finish by proposing potential and new directions of research to address noradrenergic dysfunction in AD and PD.

## Anatomical and neurobiological considerations

### Alzheimer's disease

Alzheimer's disease (AD) is a well-known cause of dementia that is associated with the accumulation of intraneuronal neurofibrillary tangles (NFTs; Braak et al., 1994) and extraneuronal neuropil threads (Perry et al., 1991). NFTs are composed of abnormally phosphorylated tau, a protein supporting cytoskeletal structure in neurons (Delacourte and Defossez, 1986; Bancher et al., 1989; Braak et al., 1994). Neuropil threads (NTs) are composed of tau and ubiquitin and located typically at distal dendrites (Perry et al., 1991). The progression of AD, first detailed by Braak and Braak in 1995, was described in six stages that correlate with accumulation of NFTs and NTs. Stage I and II are regarded as the entorhinal stage and associated with accumulation of NFTs and NTs in the transentorhinal regions (Jellinger et al., 1991; Bancher et al., 1993; Braak et al., 1994). Stage III and IV are known as the limbic stage and progress to involve considerable portions of the entorhinal cortex. The limbic stage is also associated with minor hippocampal change. Clinically, stage III/IV are associated with impaired cognitive functioning and minor personality changes (Jellinger et al., 1991; Bancher et al., 1993; Braak et al., 1994). The last two stages, stage V and VI, represent the isocortical stages of AD. The feature that distinguishes the isocortical stages is significant involvement of the cerebral cortex and hippocampus. Stage V and VI manifest in clinically diagnosable AD.

It is important to note that AD does not follow a uniform pattern of neuroanatomical changes. A more recent study of AD pathology revealed three NFT distribution patterns described as typical AD, hippocampal-sparing AD and limbic predominant AD (Whitwell et al., 2012). Typical AD presents with diffuse NFT deposition in the hippocampus and cortex. Hippocampal-sparing AD demonstrates less NFT deposition in the hippocampus but more in the cortex, when compared to typical AD. Lastly, limbic predominant AD involves significantly more NFT deposition in the hippocampus than either of the other two groups. Thus, AD appears to be a relatively heterogeneous disease in terms of its etiological processes as characterized by chemical neuroanatomy.

Whereas clinical staging focuses on changes in the cerebral cortex and hippocampus, other studies have implicated changes in the locus coeruleus (LC) and nucleus basalis of Meynert (NbM). The loss of cholinergic neurons in the NbM was demonstrated as early as 1981 by Nissl-stained histological sections and cell counts in post-mortem studies of AD (Whitehouse et al., 1981). Patients with AD demonstrated degeneration of >75% of the neurons within the NbM (Whitehouse et al., 1982). The NbM cholinergic neurons project throughout the brain and loss of the cholinergic inputs may account for behavioral changes such as short-term memory loss, disorientation and problems with language in AD (Burns and Iliffe, 2009). Other studies demonstrated significant loss of LC neurons in AD (Matthews et al., 2002; Szot et al., 2006; Insua et al., 2010). In fact, LC degeneration has been documented in AD too as early as 1981 (Tomlinson et al., 1981). In comparative studies of disease burden in patients with advanced-stage AD the NbM demonstrated the most significant cell loss followed by the LC and transentorhinal cortex (Arendt et al., 2015). LC degeneration also occurs during healthy aging, which could amount to 50% of cell loss in the tenth decade of life (Manaye et al., 1995). In a longitudinal clinical-pathologic cohort study, 165 individuals completed a battery of cognitive tests and underwent brain autopsy upon death to examine neuronal density of the LC and other aminergic nuclei in the midbrain/brain stem (Wilson et al., 2013). Modeled together, only LC neuronal density was related to cognitive decline, suggesting that the LC may be a structural component of neural reserve.

### Parkinson's disease

Structural brain changes in Parkinson's Disease (PD) similarly follow a time course. PD is associated with the formation of intraneuronal aggregates of the protein alpha synuclein, known as Lewy bodies. The accumulation of Lewy bodies is closely associated with the degeneration of the ventrolateral substantia nigra including specifically the pars compacta (SNc; Fearnley and Lees, 1991; Damier et al., 1999). The loss of SNc neurons results in diminution of extranigral dopaminergic projections throughout the striatum, allocortex, and neocortex (Braak et al., 2003).

As with AD, post-mortem pathology has been described in six different stages which were determined using alpha-synuclein-immunopositive Lewy neurites and Lewy bodies to track disease progression in PD (Braak et al., 2003). Stage 1 is associated with lesions in the dorsal IX/X motor nucleus. Stage 2 includes the findings of stage 1 with additional involvement of the caudal raphe nuclei, gigantocellular reticular nucleus, and coeruleus-subcoeruleus complex. Stage 3 is characterized by significant lesions in the midbrain specifically in the area of the SNc. Progression to stage 4 represents first cortical involvement specifically in the transentorhinal region and allocortex. Stage 5 involves the neocortex with lesions present in sensory association areas and prefrontal regions. Finally, progression to stage six is characterized by additional lesions in sensory association areas and new lesions in the premotor, primary sensory, and primary motor areas. In summary, lesions in PD start at the dorsal motor nucleus and progress to involve the LC, SNc, transentorhinal and cortical regions, in that order. It is worth noting that Braak staging of PD implies an ascending propagation of pathology similar to that of prion diseases and offers to explain the neural processes underlying symptom progression. However, the exact mechanisms of stage progression remain unclear. In particular, studies have reported that only about half of postmortem brains in PD demonstrated Lewy body pathology consistent with the Braak staging (Kalaitzakis et al., 2008; Jellinger, 2009; Halliday et al., 2012), challenging the notion that PD progresses in a sequence similar to prion diseases (Surmeier et al., 2017).

Thus, whereas degeneration of the SNc is the most classic neuropathological finding in PD, postmortem studies also demonstrate Lewy body burden and associated loss of noradrenergic neurons in the LC (Chan-Palay and Asan, 1989; Braak et al., 2006; Szot, 2012). Interestingly, the loss of LC neurons in PD has been documented to occur earlier and in greater magnitude than that of the SNc (Rommelfanger and Weinshenker, 2007). For instance, in Braak staging of PD involvement of the LC starts in stage 2 while involvement of the SNc is not present until stage 3. Loss of LC neurons in PD is associated with depletion of noradrenergic inputs in the frontal cortex, cerebellum, striatum, thalamus, and hypothalamus (Kish et al., 1984; Shannak et al., 1994; Pavese et al., 2011), all of which receive projections from the LC. Together, although postmortem anatomical studies have focused on changes in the SNc, there is significant evidence in support of changes of the noradrenergic system as a critical etiological process of PD. Table 1 highlights the anatomical and neurobiological changes in AD and PD.

**Table 1:** Summary of anatomical changes in Alzheimer's Disease and Parkinson's Disease.

**Anatomical Changes in AD and PD**	
AD	-Braak Staging (Braak et al., 1994) I/II—NFTs and NTs accumulate in transentorhinal regions III/IV—pathology in entorhinal cortex with hippocampal change V/VI—significant accumulation in cerebral cortex and hippocampus -Early loss of neurons is also seen in NbM and the LC (Szot et al., 2006)
PD	-Accumulation of LBs is closely associated with degeneration of the SNc (Fearnley and Lees, 1991; Damier et al., 1999) -Loss of SNc neurons is associated with diminution of extranigral dopaminergic projections through striatum, allocortex, and neocortex (Braak et al., 2003) -Loss of LC neurons occurs earlier and in greater magnitude than that of the SNc (Rommelfanger and Weinshenker, 2007) -Loss of LC neurons is associated with depletion of noradrenergic inputs in the frontal cortex, cerebellum, striatum, thalamus, and hypothalamus (Kish et al., 1984; Shannak et al., 1994; Pavese et al., 2011)

*AD, Alzheimer's Disease; PD, Parkinson's disease; NFT, neurofibrillary tangle; NT, neuropil thread; NbM, nucleus basalis of Meynert; LC, locus coeruleus; LB, Lewy body; SNc, substantia nigra pars compacta*.

## Structural brain imaging in AD and PD

### Alzheimer's disease

Many studies have correlated Braak staging with atrophic changes on magnetic resonance imaging (MRI) using voxel-based morphometry (VBM) (Ohnishi et al., 2001; Matsuda et al., 2002; Chetelat et al., 2003; Rémy et al., 2005; Matsuda, 2013), focusing largely on hippocampal atrophy as a biomarker of AD (Jack et al., 2011). The patterns of cerebral atrophy as detected on MRI vary across pathological subtypes of AD (Whitwell et al., 2012; Noh et al., 2014). Medial temporal cortical atrophy is seen in limbic-predominant while severe cortical atrophy is seen more in hippocampal-sparing AD (Whitwell et al., 2012). Distinct anatomical changes have also been identified on MRI when early and late onset AD are contrasted (Frisoni et al., 2007). Individuals with early onset AD tend to demonstrate more extensive occipital gray matter atrophy whereas those with late onset AD demonstrate more extensive hippocampal atrophy. The notion of prioritizing anatomical integrity of an isolated region as a biomarker of AD may be convenient; however, the anatomical heterogeneity brings to question the sensitivity of structural changes of individual brain regions as a diagnostic tool of AD.

Although the LC is implicated by postmortem anatomical studies, only recently have studies begun to employ high-resolution fast spin-echo T1-weighted imaging to reveal signal attenuation of the LC in individuals with mild cognitive impairment (MCI) and AD (Takahashi et al., 2015). This finding corresponds well to pathological findings of a decrease in neuromelanin contents as a result of LC neuronal loss in these patients. It is important to note that conventional MRI has not been successful in delineating the LC. However, fast spin-echo T1-weighted imaging, which relies on the paramagnetic properties of neuromelanin, has been demonstrated as a reliable method to quantify signal attenuation or volume loss in neuromelanin- containing tissues such as the LC and SNc (Tosk et al., 1992; Enochs et al., 1997). Neuromelanin is a pigment found in both SNc and LC (Lehéricy et al., 2012). Neuromelanin is identified in monoamines-containing neurons and formed by polymerization of 4,5-dihydroxyindole monomers (Charkoudian and Franz, 2006) via enzymatic processes that involve monoamine oxidase (Rabey and Hefti, 1990), and its concentrations increase with age. Neuromelanin can chelate metals and protect against oxidative stress (Tribl et al., 2009). On the other hand, excessive neuromelanin accumulation compromises neuronal integrity and causes dissolution of cell mass and decrease in neuromelanin signals, as may occur in neurodegenerative conditions. Neuromelanin has paramagnetic-T1 shortening effects and, when combined with metals like iron and copper, would make the SNc and LC appear hyper-intense on MRI. On neuromelanin-MRI there is signal attenuation in MCI participants who do or do not to progress to AD, suggesting that attenuated LC signal alone may not be diagnostic of AD. In fact, the LC exhibited signal changes during healthy aging (Shibata et al., 2006; Betts et al., 2017). Most recently, a stereological study of postmortem human brains reported that as the Braak staging increases by 1 unit, LC volume decreases by 8.4% (Theofilas et al., 2017). Together, although not necessarily pathognomonic of AD, LC volume measurements offer a promising biomarker to track the progression to AD from presymptomatic stages.

### Parkinson's disease

Findings from VBM studies of PD demonstrate varying results with the most common changes identified in the frontal and parieto-occipital regions (Biundo et al., 2011; Pan et al., 2012; Lee et al., 2013; Fioravanti et al., 2015). A meta-analysis of 498 patients with idiopathic PD revealed reductions in gray matter (GM) volume in the left frontal temporal cortices encompassing inferior frontal and superior temporal gyri (Pan et al., 2012). Other brain areas demonstrating GM reductions in PD were the left insular cortex, with insular GM density potentially related to memory scores (Pan et al., 2012; Lu et al., 2016), and the middle and superior frontal gyrus (Biundo et al., 2011). In a study where patients were scanned twice 2 years apart, PD but not controls demonstrated significant GM loss in the putamen and parietal cortex (Fioravanti et al., 2015). On the other hand, some studies did not reveal any significant differences in GM volume between PD and healthy controls (Menke et al., 2014; Borroni et al., 2015). In one of these studies PD patients were split into those with and without dementia and the PD group with dementia but not the group without demonstrated reductions in frontal regional GM volume (Borroni et al., 2015). However, compared to health controls, the two groups combined did not show frontal GM reduction. Together, these findings suggest that variability across studies may relate to the cognitive status of study participants. Other issues including sample size, duration of illness, medication status may all impact the imaging findings.

Neuromelanin Imaging has also been widely used to examine the integrity of SNc function in PD (Shibata et al., 2006; Ohtsuka et al., 2014; Castellanos et al., 2015; Reimão et al., 2015). In melanin-sensitive MR sequences both the SNc and the LC demonstrate reduction in signal intensity or contrast ratio (CR) in PD (Sasaki et al., 2006). The finding of decreased neuromelanin CR in the SNc has been reported in multiple studies of PD (Sasaki et al., 2006; Matsuura et al., 2013; Miyoshi et al., 2013; Mukai et al., 2013; Ohtsuka et al., 2014; Isaias et al., 2016). A recent work of neuromelanin-sensitive MRI employed an automated segmentation algorithm to quantify the volumes of the SNc and LC (Castellanos et al., 2015). Receiver operating characteristic analysis demonstrated better accuracy using the SNc than LC volumes in the diagnosis of PD. Neuromelanin-sensitive MRI has also been used to differentiate essential tremor from PD (Reimão et al., 2015) and early-stage PD from healthy controls (Ohtsuka et al., 2014). Thus, neuromelanin MRI offers a promising technique for characterizing structural and potentially functional changes of the SNc and LC in PD.

### Summary

Neuromelanin-sensitive MRI has become a popular tool to investigate LC function and dysfunction. Neuromelanin CRs enable better visualization and assessment of the anatomical and functional integrity of the LC. In healthy adults neuromelanin CR of the LC increased until the sixth decade of life at which point it started to decrease (Shibata et al., 2006). This as well as another study (Clewett et al., 2016) suggests that LC neuromelanin accumulates in an inverted U pattern with age and peaks around 60 years. In older adults, greater LC signal intensity was associated with higher composite scores of cognitive reserve (Clewett et al., 2016). Neuromelanin CRs were greater in healthy adults as compared to patients with major depression, schizophrenia, MCI, PD, and AD (Liu et al., 2017). Together, these findings demonstrate the importance of LC imaging in investigating cognitive changes in neurodegenerative conditions and other psychiatric illnesses that implicate monoaminergic dysfunction.

## Functional brain imaging in AD and PD

Functional MRI (fMRI) utilizes blood-oxygenation level dependent (BOLD) signals as a proxy for neural activity. The premise of using BOLD signals to reflect neural activities lies in the fact that oxyhemoglobin and deoxyhemoglobin exhibit different magnetic properties. As neurons are activated, more oxygen is consumed, resulting in a change in the ratio of oxyhemoglobin and deoxyhemoglobin, which can be picked up by BOLD imaging. Many studies have combined fMRI and a behavioral task to examine cerebral responses to cognitive and affective challenges. However, a decade of work has suggested that functional organization of the brain can be delineated by how BOLD signals of brain regions are correlated during a resting state. Low-frequency BOLD signal fluctuations reflect connectivity between functionally related brain regions (Biswal et al., 1995; Fair et al., 2007; Fox and Raichle, 2007). Studies of this “spontaneous” activity have provided insights into the intrinsic functional architecture of the brain and shown that coordinated spontaneous fluctuations are present in motor, visual, auditory, default mode, memory, language, dorsal attention, and ventral attention systems (Fox and Raichle, 2007). Other studies have suggested connectivity analysis of resting-state fMRI data as a useful alternative to characterizing functional subdivisions of a brain region (Zhang and Li, 2012a,b, 2014, 2017).

In a study of resting state functional connectivity (rsFC), the LC demonstrated positive connections to bilateral superior frontal gyrus, primary motor cortex, inferior parietal cortex, inferior temporal cortex, anterior parahippocampal gyrus, posterior insula, putamen, pallidum, ventrolateral thalamus, midbrain, and large areas of the cerebellum (Zhang et al., 2016). The LC showed negative connectivities too to a wide swath of brain regions, including bilateral visual cortex, middle/superior temporal cortex, precuneus, retrosplenial cortex, posterior parahippocampal gyrus, frontopolar cortex, caudate nucleus, and the dorsal and medial thalamus. Findings of positive connectivity support a role for norepinephrine (NE) in orienting and sensorimotor responses to external stimuli. The findings of negative connectivity to the precuneus support a role for the LC in regulating activity of the default-mode network (DMN). Together, through connectivity to the cerebral cortex the LC may suppress DMN activity in response to external stimuli and facilitate engagement of the saliency (“alert and orient”) and executive control systems (Li et al., 2007; Zhang and Li, 2010, 2012a,b). On the other hand, one should be cautioned against over-interpreting rsFC in terms of functional implications. A positive rsFC does not necessarily mean that the two brain regions are functionally congruent. Long range cortical-cortical/subcortical projections may target inhibitory interneurons within the recipient regions (Thomson and Lamy, 2007; Apicella et al., 2012; DeNardo et al., 2015). For instance, the amygdala and ventromedial prefrontal cortex (vmPFC) show positive rsFC (Veer et al., 2010; Schultz et al., 2012) but may respond in isolation or in opposite directions to behavioral challenges such as affective regulation (Urry et al., 2006) and extinction learning of fear (Phelps et al., 2004).

The proposition that the LC circuit responds to behavioral tasks is supported by task-based studies demonstrating LC activation to stimulus change. In an oddball task LC activity increased to the detection of novel, oddball stimuli (Krebs et al., 2017). In a picture-word interference task the LC responded to novel as compared to familiar stimuli (Krebs et al., 2013). In an attention task, exposure to the alerting condition was associated with increased activation of the LC (Neufang et al., 2015). The LC is also consistently activated in response to stimuli that trigger arousal responses via fear, pain, and anger (Liddell et al., 2005; Brooks et al., 2017; Gilam et al., 2017). The role of the LC in arousal is examined by a recent study using dexmedetomodine to elicit a state of reduced arousal (Song et al., 2017). Dexmedetomidine, a potent sedative, is an α2 agonist that reduces LC neuronal firing and NE release. Along with decreasing arousal, dexmedetomidine diminished connectivity between the LC and posterior cingulate cortex (PCC) as compared to the awake state. Further, dexmedetomidine disrupted PCC connectivity with subcortical and cortical structures central to executive control. These findings together support the role of the LC in facilitating attention shifts in response to stimulus change and in situations that demand a higher level of alertness (see Liu et al., 2017 for a review of task-related LC studies).

Finally, an overview of electrophysiological studies of LC neuronal functions would place human imaging findings in a clearer perspective (Aston-Jones and Waterhouse, 2016). First, cortical projections of the LC are predominantly ipsilateral whereas subcortical projections are more likely to be bilateral. Further, projections of individual LC neurons collateralize to cover functionally related brain regions. Neurons projecting to different circuits are segregated in the LC and may release NE asynchronously in response to task demands. Second, studies employing iontophoresis of NE in target regions and electrical stimulation of the LC showed that LC suppresses spontaneous neuronal activity in the cerebellum and cerebral cortex, establishing NE as an inhibitory neurotransmitter at central synapses. Other studies showed that the spontaneous activities are suppressed to a greater extent than stimulus-evoked activity, resulting in an increase of the signal noise ratio in response to stimulus. Third, LC neurons discharge both tonically and phasically. LC neurons discharge tonically at a moderate rate but respond to task-related stimuli phasically during focused attention. The Adaptive Gain Theory proposes that LC phasic responses facilitate behavioral adaptation to changing environment whereas tonic activities suppress behavior of low utility (Aston-Jones and Waterhouse, 2016).

### Functional imaging studies of AD

The default mode network (DMN) comprises a set of brain regions, including parts of the precuneus and posterior cingulate cortex (PCC; Leech and Sharp, 2013), that are more active during mind wandering, retrieval of autobiographical memories, and monitoring of the arousal state (Leech and Sharp, 2013), than during exposure to environmental stimuli. A host of studies have noted changes in DMN activity and connectivity in AD during rest (Jacobs et al., 2013; Badhwar et al., 2016; Kim et al., 2016). Reduced DMN connectivities can be observed from early stages of MCI to late stage AD (Adriaanse et al., 2014; Gour et al., 2014; Badhwar et al., 2016), as well as in asymptomatic patients genetically at risk of AD (Chhatwal et al., 2013; Sala-Llonch et al., 2013; Quiroz et al., 2015). Reduced DMN connectivity in AD also correlates with disease severity, as measured by the Clinical Dementia Rating scale (Zhou et al., 2010; Petrella et al., 2011; Brier et al., 2012). Of all the brain networks studied, the DMN demonstrates the most consistent connectivity changes in AD.

The salience network (SAN) is a network of structures including most prominently the dACC and anterior insula that processes a constant stream of external stimuli to identify salient inputs for goal-directed behavior. In contrast to the DMN, which is an inwardly driven network, the SAN integrate sensory, visceral, and autonomic information to facilitate decision making (Seeley et al., 2007). Changes in the SAN activity and connectivity are also implicated in AD. Network based analyses frequently demonstrate hyperconnectivity within the SAN in patients with AD (Thomas et al., 2014; Wang et al., 2015; Badhwar et al., 2016). Abnormal SAN connectivity has been documented across the disease spectrum from early to late onset AD (Wang et al., 2015; Badhwar et al., 2016), in patients with autosomal dominant AD (Thomas et al., 2014), and in those who carry the APOEe4 gene, which is known to increase the risk for AD (Machulda et al., 2011; Goveas et al., 2013). One of the key hubs of the SAN is the anterior insular cortex (Seeley et al., 2007). In a large, voxel-based meta-analysis, the anterior insula demonstrated significant hyperconnectivity in AD (Badhwar et al., 2016). The SAN receives limbic and autonomic inputs and facilitates activity shifts between the DMN and frontoparietal network to help guide behavior (Seeley et al., 2007; He et al., 2014). Moreover, changes in SAN connectivity may perturb DMN function and vice versa (Bonnelle et al., 2012; Zhao Q. et al., 2017). Thus, the SAN plays a critical role in regulating DMN activity and failure to suppress DMN activity during task challenges correlates with worse cognitive performance. The implications of these changes for AD will be discussed in more depth later.

Other studies demonstrated hyperconnectivity of the limbic network (Gour et al., 2011, 2014; Badhwar et al., 2016), mostly in the hippocampus and entorhinal cortex, in AD (Badhwar et al., 2016). Intriguingly, anterior temporal network hyperconnectivity was correlated positively with memory performance in patients with early onset AD (Gour et al., 2014), perhaps reflecting a transient compensation before progression to late-stage dementia. Together with findings on the DMN, network connectivity changes appear to be a prominent feature of AD. The significance of these findings needs to be evaluated with longitudinal records of disease progression.

### Functional imaging studies of PD

In patients with PD slower processing speeds were associated with decreased DMN connectivity at rest, specifically between the posterior cingulate, medial prefrontal and inferior parietal nodes (Disbrow et al., 2014). Off medication, PD patients were unable to suppress DMN activity during a facial emotion recognition task (Delaveau et al., 2010). In the Montreal card-sorting task, a task that involves manipulation of short-term memory, patients with PD showed less deactivation of the DMN as compared to healthy controls (van Eimeren et al., 2009). Another study reported worse performance on executive functioning, psychomotor speed and verbal memory in association with increased positive connectivity between the SAN and DMN (Putcha et al., 2016). In other words, reduced anti-correlation between these two networks was associated with impaired cognitive performance in PD. The latter study implicates dysregulation of both SAN and DMN in PD.

The right inferior parietal cortex (IPC), a region associated with the DMN (Mars et al., 2012; Davey et al., 2016), demonstrated aberrant connectivity in PD patients (Tahmasian et al., 2017) with and without depression (Wen et al., 2013), particularly in those who were cognitively impaired (Amboni et al., 2015; Gorges et al., 2015). Altered IPL activity was demonstrated consistently across different neural metrics, including the amplitude of low frequency fluctuations and regional homogeneity (Tahmasian et al., 2017). In a meta-analysis of rs-fMRI studies, connectivity changes in the right posterior IPC represent the most consistent finding in PD (Tahmasian et al., 2017) and may disrupt activity of the precuneus, PCC, mid-cingulate cortex, middle and superior frontal gyrus, and orbitofrontal gyrus. It is worth noting again that, the posterior IPC is part of the DMN (Davey et al., 2016). Sub-cluster analysis of functional connectivity identified four functionally dissociable subregions in the IPC (Zhang and Li, 2014; Bzdok et al., 2015) and the posterior subregion, in the area of angular gyrus and with the strongest connectivity to the DMN, is the exact region that demonstrated aberrant connectivity in PD (Tahmasian et al., 2017). PD patients demonstrated increased parietal cortical activation when off as compared to on medication (Herz et al., 2014). Mutation carriers of leucine-rich repeat kinase 2 (LRR2), a population with an elevated risk for PD, also demonstrated reduced connectivity between the IPL and posterior putamen, a task network region (Helmich et al., 2015). Altered DMN activity was associated with decreased SNc activity and with disease severity in PD (Wu et al., 2012). In summary, as with AD, dysconnectivity of the DMN appears a core neural feature and may contribute to cognitive decline in PD.

## Other imaging studies

Conventional T1/T2 MRI has not been successful at identifying changes in the SNc or other structures in PD. More advanced techniques including magnetization transfer (Wolff and Balaban, 1989; Rademacher et al., 1999; van Waesberghe et al., 1999; Helms et al., 2009), adiabatic and MR microscopy (Lehéricy et al., 2012) and relaxometry have revealed changes in the SNc in individuals with PD. In addition to neuromelanin imaging, iron imaging has been studied in PD. Brain iron content is increased in association with dopamine loss in the SNc in PD patients (Dexter et al., 1991; Martin et al., 2008; Martin, 2009; Mascalchi et al., 2012). Thus, imaging iron with nonionizing MRI offers a strategy to detect neuroanatomical changes in PD. In a two-year follow-up study iron-related relaxation increased in the anterior globus pallidus, caudate nucleus and medial SNc and the changes in the globus pallidus and SNc were related to the development of MCI (Rossi et al., 2014). More recently an iron imaging study employing quantitative susceptibility mapping (QSM) reported increased QSM magnetic values in PD patients as compared to healthy controls. The difference between SNc iron deposition between healthy controls and patients with advanced PD was the most prominent, with SNc iron content correlated with symptom severity in PD (An et al., 2018).

Positron emission tomography (PET) imaging of PD focused primarily on changes in dopamine transporter (DAT) density. In a recent meta-analysis DAT and vesicular monoamine transporter in early to moderate PD is decreased most significantly in the posterior putamen followed by the anterior putamen and caudate. Moreover, disease severity was linearly correlated with dopamine loss (Kaasinen and Vahlberg, 2017). No studies to our knowledge investigated norepinephrine transporter (NET) density in PD. [(11)C]MENNET is a novel PET radiotracer with high affinity and selectivity for NET and may provide insights into noradrenergic dysfunction in PD (Adhikarla et al., 2016).

PET imaging has also demonstrated utility in tracking AD progression. Fluorine-18 fluorodeoxyglucose (FDG) PET imaging assessed regional cerebral glucose metabolism and showed decreased metabolic rates in the medial temporal lobes, lateral temporoparietal cortex, posterior cingulate cortex, and precuneus in patients with AD in comparison to normal aging (Sarikaya, 2015). In a post-mortem autoradiographic study of (S,S)-[(18)F]FMeNER-D(2), a selective ligand for NET, AD patients showed a reduction in NET density at the LC and thalamus in correlation with disease progression by Braak staging (Gulyás et al., 2010). The size of the LC is below the spatial resolution of PET imaging but *in vivo* imaging of NET density in the thalamus may be useful to highlight early noradrenergic dysfunction in AD (Gulyás et al., 2010).

Table 2 highlights VBM, neuromelanin and other imaging findings of AD and PD.

**Table 2:** Summary of imaging findings in Alzheimer's Disease and Parkinson's Disease.

**Non-Functional Imaging in AD and PD**		
AD	Voxel-based morphometry	Hippocampal atrophy (Jack et al., 2011)
		Medial temporal atrophy in limbic-predominant AD (Whitwell et al., 2012)
		Severe cortical atrophy in hippocampal-sparing AD (Whitwell et al., 2012)
		More extensive occipital GM atrophy in early- vs. late- onset AD (Frisoni et al., 2007)
		More extensive hippocampal atrophy in late- vs. early-onset AD (Frisoni et al., 2007)
	Neuromelanin Imaging	LC demonstrates neuromelanin signal attenuation in MCI (Shibata et al., 2006; Betts et al., 2017)
		LC volume decreases by 8.4% with progression to each consecutive Braak stage, as measured by neuromelanin signals (Theofilas et al., 2017)
	PET Imaging	PET imaging with F^18^-FDG radioligand reveals decreased cerebral metabolic rates in the medial temporal lobes, lateral temporoparietal cortex, posterior cingulate cortex and precuneus (Sarikaya, 2015)
		(S,S)-[(18)F]FMeNER-D(2), a radioligand specific for norepinephrine transporter (NET), demonstrates reduced NET density in the LC and thalamus on postmortem brains (Gulyás et al., 2010)
PD	Voxel-based morphometry	GM volume reductions in the left frontal temporal cortices encompassing inferior frontal and superior temporal gyri (Pan et al., 2012)
		GM reductions in left insular cortex (Pan et al., 2012; Lu et al., 2016)
		PD patients with dementia have more prominent reductions in frontal regional GM (Borroni et al., 2015)
	Iron Imaging	Brain iron content in the SNc is increased in PD patients, in association with loss of DA neurons (Dexter et al., 1991; Martin et al., 2008; Martin, 2009; Mascalchi et al., 2012)
		Increased iron-content in the globus pallidus and anterior and medial SNc, in correlation with MCI in PD (Rossi et al., 2014)
		Iron content in the SNc as measured by quantitative susceptibility mapping correlates with the symptom severity of PD (Liu et al., 2017)
	Neuromelanin Imaging	SNc and LC demonstrate reduction in signal intensity in PD (Fox and Raichle, 2007; Zhang and Li, 2012a,b, 2014, 2017; Zhang et al., 2016)
		Differences on neuromelanin-sensitive MRI distinguish essential tremor from PD and early-stage PD from healthy-controls (Fair et al., 2007; Fox and Raichle, 2007)
	PET Imaging	Decline in dopamine transporter occurs most significantly in the posterior putamen followed by anterior putamen and caudate and there is a correlation between dopamine loss and disease severity (Kaasinen and Vahlberg, 2017)

*AD, Alzheimer's Disease; PD, Parkinson's disease; MCI, Mild cognitive impairment; GM, gray matter; LC, locus coeruleus; PET, positron emission tomography; F^18^-FDG, Fluorine-18 fluorodeoxyglucose; NET, norepinephrine transporter; GM, gray matter; SNc, substantia nigra pars compacta; DA, dopamine*.

## Noradrenergic dysfunction in AD and PD

### An overview

The LC sends noradrenergic projections to the hippocampus (Loughlin et al., 1986), amygdala (Fallon et al., 1978), and prefrontal cortex (PFC; Loughlin et al., 1982). Phasic LC activation in response to target stimuli facilitates anticipation (Aston-Jones et al., 1985, 1994) and release of norepinephrine (NE) in the cortex (Mountcastle et al., 1972; Aston-Jones and Cohen, 2005) prior to a motivated action. NE signals in the PFC regulate attention, learning and working memory (Robbins, 2000). On the other hand, NE interacts with other catecholamines like dopamine (DA) to support these functions, with NE often playing a regulatory role in DA signaling. For example, chemical modulation or electrical stimulation of the LC increases the extracellular concentrations of both NE and DA (Smith and Greene, 2012). The ability of the LC to effect direct and indirect control of catecholamines has important implications on the arousal state as well as autonomic, motor, sensory, and cognitive functions.

Both AD and PD involve noradrenergic dysfunction. For example, orthostatic and postprandial hypotension in PD can be related to autonomic dysfunction and NE deficiency (Kaufmann and Goldstein, 2013). Disruption of the circadian rhythm and arousal/wakefulness cycles manifest in both diseases and are associated with noradrenergic dysfunction (Berridge and Waterhouse, 2003; Benarroch, 2009). In PD, decreased CSF concentration of NE is associated with freezing of gait and administration of a NE precursor improves gait (Tohgi et al., 1993a,b). Animal studies involving degeneration of the LC also support noradrenergic dysfunction in AD. In transgenic mice carrying homozygous forms of amyloid precursor protein/presenilin 1 genes, induction of LC degeneration with N-(2-chloroethyl)-N-ethyl-bromo-benzylamine resulted in an exacerbation of olfactory memory deficits and weakening of olfactory discrimination (Rey et al., 2012). As 90% of patients with early AD experience olfactory dysfunction (Hawkes, 2003), the latter study suggests a potential role of the LC in the etiological processes of early AD. In another study of transgenic mice with a low amyloid load, LC degeneration elicited with the same chemical triggered massive glial inflammation and augmented the deposition of amyloid plaques (Heneka et al., 2006). Together, these studies provide substantial evidence for noradrenergic dysfunction in AD and PD.

### Noradrenergic dysfunction and the hippocampus in AD and PD

AD and PD are known to have distinct pathology, yet they share many cognitive and behavioral manifestations as the illness progresses, including dementia, motor dysfunction, and behavioral disinhibition. Both AD and PD are associated with significant degeneration of the LC (Bondareff et al., 1982; Marcyniuk et al., 1986; Rommelfanger and Weinshenker, 2007). In PD the degeneration of the LC occurs earlier and in greater magnitude in comparison to the SNc (Rommelfanger and Weinshenker, 2007). AD and PD also share extensive pathology in the hippocampus. In AD, memory deficits tend to manifest early and correlate with hippocampal pathology (Braak et al., 1994). In PD, loss of NE-containing neurons is associated with cognitive decline (Cash et al., 1987). As the LC projects heavily to the hippocampus and parahippocampal formation (Zhang et al., 2016), loss of noradrenergic signaling may lead to hippocampal dysfunction and memory deficits. In patients with MCI and a Clinical Dementia Rating score of 0.5, decreased LC connectivity to the left parahippocampal gyrus was associated with lower memory performance (Jacobs et al., 2015). These findings support the proposition that noradrenergic dysfunction in AD and PD contributes to memory impairment.

In rodent studies, the LC has extensive anatomical connectivity (likely stronger than the ventral tegmental area) to the hippocampus (Takeuchi et al., 2016). Optical activation of LC terminals in the dorsal hippocampus enhances object location memory (Kempadoo et al., 2016). Further, depletion of noradrenergic terminals in the hippocampus by neurotoxics or ablation of LC neurons impaired location memory in mice (Coradazzi et al., 2016; Moreno-Castilla et al., 2017). Numerous other studies have implicated noradrenergic control of other aspects of cognitive functioning (Caetano et al., 2013). Reduced cortical noradrenergic neurotransmission was associated with increased neophobia and impaired spatial memory in aged rats (Collier et al., 2004). Work on transgenetics associated reduced tissue levels of NE and LC degeneration with behavioral phenotypes of mouse models of AD (Francis et al., 2012; Rey et al., 2012). In transgenic mice that accumulated amyloid burden at early ages, treatment with a NE precursor increased CNS NE levels and improved learning in the Morris water maze (Kalinin et al., 2012). Noradrenergic innervations from the LC are needed to maintain beta amyloid clearance, and LC degeneration could contribute to the pathogenesis of AD (Kalinin et al., 2007).

The potential roles of NE in the etiological processes of AD and PD and memory impairment can also be examined with pharmacologic manipulations. Administration of methylphenidate, a stimulant medication known to increase NE and DA levels and commonly used in the treatment of ADHD (Weikop et al., 2007), improved word recall when taken before learning (Verster et al., 2010; Linssen et al., 2012). Administration of methylphenidate after learning also increased 1-week retention of intentionally and casually learned information (Izquierdo et al., 2008). In a fMRI study, methylphenidate administration increased connectivity between the LC and the hippocampus (Kline et al., 2016). The latter study implicates NE in producing a measurable change in connectivity between the LC and hippocampus, both of which suffer significant neuronal loss and functional dysconnectivity in AD and PD. Although methylphenidate increases the extracellular levels of DA in addition to NE, the finding of increased connectivity between the LC and hippocampus and its association with improved memory function helps establish a specific link to the noradrenergic system. Together, considered in the context of LC degeneration and the resulting denervation of the hippocampus, these findings support noradrenergic dysfunction as a neural mechanism of memory impairment in AD and PD.

### Noradrenergic connectivity dysfunction in AD and PD

The LC undergoes early and significant deterioration and recent imaging studies suggest LC functional dysconnectivity that involves brain regions other than the hippocampus in AD. A recent work employed Granger causality mapping to create a model of directed connectivity and characterized regional functional coupling in AD and healthy controls (Zhao S. et al., 2017). With group differences determined with a generative model of pathology, the authors identified the LC and right orbitofrontal cortex as the two foci of disruption in AD. These findings provide further support of a critical role of the LC in functional impairment in AD.

The saliency network (SAN) facilitates network activity transition from the DMN to the frontoparietal network during task challenges (Seeley et al., 2007; He et al., 2014). For instance, the right anterior insula temporally precedes activation of the central executive network and de-activation of the DMN in task based paradigms (Sridharan et al., 2008). Temporal control over these network activities is central to an intact cognition, and impaired suppression of the DMN is associated with impaired performance. As discussed earlier, abnormal DMN connectivity represents one of the most consistent finding in AD. The SAN, specifically the anterior insula, also demonstrates abnormal connectivity in AD. DMN and SAN dysfunction may result in large part from noradrenergic dysfunction in AD. The insula responds strongly to deviances in a stream of continuous stimuli and does so across auditory, visual, and tactile modalities (Linden et al., 1999; Downar et al., 2000, 2001; Crottaz-Herbette and Menon, 2006). Regions of the SAN coactivate in response to faces of loved ones (Bartels and Zeki, 2004), pleasurable touch (Craig, 2002), emotional dimensions of pain (Peyron et al., 2000), “chills” to music (Blood and Zatorre, 2001) and other forms of saliency (Eisenberger et al., 2003; Singer et al., 2004), all supported by LC activity and an intact system of physiological arousal. As the LC shores up arousal and responses to salient stimuli, noradrenergic signaling is in a position to influence SAN and DMN network activities. It is highly likely that deficient noradrenergic signaling may compromise functional coupling between the SAN and DMN and, as a result, deficits in the suppression of DMN activity to meet task demands.

In support, atomoxetine, a selective NE reuptake inhibitor commonly used in the treatment of ADHD, increased connectivity between the right anterior insula and dorsolateral prefrontal cortex (Hernaus et al., 2017). Increased connectivity between these regions correlated with decreased reaction time variability in an N-back working memory test and predicted auditory digit span of the Wechsler Adult Intelligence Scale. In a predictive learning task atomoxetine facilitated extinction learning along with increased activity in the insula (Lissek et al., 2015). In a study of inhibitory control, atomoxetine improved performance in a stop-signal task in correlation with increased insula activity (Chamberlain et al., 2009). These findings suggest that NE influences insula activity and improves a broad spectrum of cognitive functions, providing a pathway whereby noradrenergic dysfunction may contribute to aberrant SAN and DMN activations in AD.

As discussed earlier, the IPL is part of the DMN (Bzdok et al., 2015) and consistently demonstrates abnormal connectivity in PD. Engagement in a cognitive task is associated with significant posterior IPL deactivation (Greicius et al., 2003; Buckner et al., 2008). Altered IPL and DMN connectivity in PD during task engagement (van Eimeren et al., 2009; Delaveau et al., 2010; Putcha et al., 2016) may represents a failure in activity transition between functional networks (Putcha et al., 2016). Successful task engagement requires suppression of internally driven processes such as mind-wondering, and the salience network monitors external stimuli and facilitates the suppression of DMN activity and these internally driven processes. One study discussed earlier demonstrated reduced anti-correlation between the SAN and DMN in link with diminished executive functioning, psychomotor speed and verbal memory (van Eimeren et al., 2009). The LC responds to saliency and functionally connects to key regions within the DMN including the IPL, PCC, and precuneus (Zhang et al., 2016). It is also one of the first brain regions affected in PD (Rommelfanger and Weinshenker, 2007). Thus, early deterioration and dysconnectivity of the LC is positioned to effect cognitive decline in PD.

Pharmacological investigations provide more evidence relating cognitive decline to LC dysfunction in PD. Enhancing noradrenergic signaling improves cognitive performance in PD (Bédard et al., 1998; Marsh et al., 2009; Weintraub et al., 2010; Obeso et al., 2011; Kehagia et al., 2014; Nombela et al., 2014; Ye et al., 2015). For example, naphtoxazine, a selective α1 agonist, as compared to placebo, ameliorated performance deficits in a reaction time task and the Stroop task in PD patients (Bédard et al., 1998). A recent study of healthy adults reported that LC functional connectivity increased in the Stroop task, implicating the noradrenergic system during interference control (Köhler et al., 2016). In other studies, atomoxetine improved performance on measures of global cognition (mini-mental status exam) and executive functions, as evaluated by the Frontal Systems Behavior Scale and Connors Adult ADHD Rating Scale (Marsh et al., 2009; Weintraub et al., 2010) in patients with PD. In a stop signal task, atomoxetine improved stop signal reaction times in a subset of PD patients (Obeso et al., 2011; Kehagia et al., 2014; Nombela et al., 2014; Ye et al., 2015). Atomoxetine also restored functional connectivity between the presupplementary motor area, inferior frontal gyrus, and subthalamic nucleus (Rae et al., 2016) of the cortical subcortical circuit to support response inhibition in PD patients. As discussed earlier, atomoxetine improved performance on stop signal tasks along with increased insular activity and engagement of the saliency network in healthy adults (Chamberlain et al., 2009). This is important because control of the DMN relies critically on SAN function (Bonnelle et al., 2012; Zhao Q. et al., 2017). In a study of medication-naïve adults with ADHD, atomoxetine facilitated suppression of the DMN in correlation with improved clinical symptoms (Lin and Gau, 2016). Together, these findings support noradrenergic dysfunction and the efficacy of noradrenergic agents in improving cognition in PD. Table 3 highlights key findings of DMN dysfunction in AD and PD.

**Table 3:** Summary of functional connectivity changes of the default mode and saliency networks in Alzheimer's disease and Parkinson's disease.

**Functional connectivity changes in AD and PD**		
AD	DMN	Reduced DMN connectivity during resting state in early stage MCI to late stage AD and in asymptomatic patients at genetic risk for AD (Seeley et al., 2007; Zhou et al., 2010; Machulda et al., 2011; Thomas et al., 2014; Wang et al., 2015)
		Reduced DMN connectivity in AD correlates with disease severity as measured by the Clinical Dementia Rating (Gour et al., 2011; Disbrow et al., 2014; Zhao Q. et al., 2017)
	SAN	Abnormal SAN connectivity across disease spectrum and those at genetic risk of AD (van Eimeren et al., 2009; Zhou et al., 2010)
		Anterior insular cortex, a key hub of the SAN, demonstrates hyperconnectivity to the SAN in AD (Delaveau et al., 2010)
PD	DMN	The most consistent connectivity change in PD involves a region of the R posterior IPC, which is part of the DMN (Rademacher et al., 1999) and altered IPC connectivity is more prominent in PD patients with cognitive impairment (Martin et al., 2008; Martin, 2009)
		Slower processing speeds in PD are associated with decreased DMN connectivity at rest, specifically between the posterior cingulate, medial prefrontal and inferior parietal nodes (Bzdok et al., 2015)
	SAN	Reduced anti-correlation between the SAN and DMN is associated with worse performance on tests of executive functioning, psychomotor speed and verbal memory (Wu et al., 2012)

*AD, Alzheimer's disease; PD, Parkinson's disease; DMN, default mode network; MCI, mild cognitive impairment; SAN, salience network; R posterior IPC, right posterior inferior parietal cortex*.

**Figure 1:**
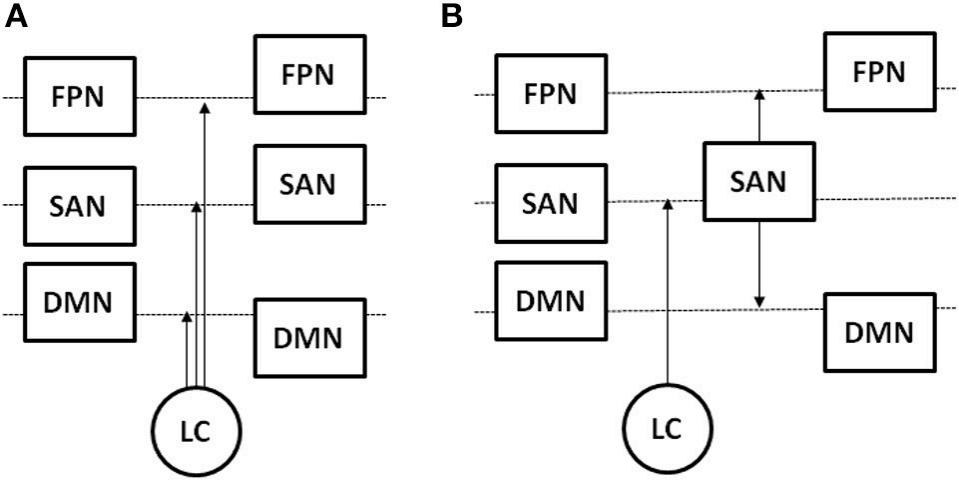
Hypothetical models of the influence of LC on the activity of the saliency network (SAN), frontoparietal task network (FPN), and default model network (DMN). Box position with respect to the dashed line represents changes in activity level from a resting to task state, and arrows indicate the location of LC action in response to a task challenge. **(A)** LC increases activity of the SAN and FPN and suppresses activity of the DMN; **(B)** LC increases activity of the SAN, which in turn increases activity of the FPN and suppresses activity of the DMN.

Figure 1 illustrate a hypothetical model of noradrenergic circuit regulation of functional neural networks.

## Conclusions and future research

Dysfunction of the noradrenergic system is a key feature of both AD and PD. The deficit is manifested in post-mortem studies which reveal loss of NE neurons in the LC in both diseases. It is also manifested in structural brain imaging as atrophic changes in structures connected with the LC and in functional imaging as changes in network activity and connectivity. Key networks implicated in noradrenergic dysfunction include the hippocampus, DMN and the SAN. Specifically, noradrenergic dysfunction may disrupt the ability to monitor external stimuli and shift attentional demands accordingly. These shifts in attentional demand rely on the SAN to regulate and suppress the DMN during task-oriented processes. Pharmacologic studies with NE reuptake inhibitors highlight the role of NE in remediating aberrations in functional connectivity and deficits in cognitive performance. Together, the studies may facilitate research of the etiology of AD and PD as well as development of pharmacotherapy for these debilitating illnesses (Calsolaro and Edison, 2015; Greig, 2015; Leissring, 2016; Schaeffer and Berg, 2017).

Despite the advances in methodology, there are limitations in current imaging techniques and pharmacological approaches to precisely localize the LC and quantify LC functions. For instance, neuromelanin imaging provides data with low spatial resolution with respect to the size of the LC and with signal non-uniformity in a given plane (Sasaki et al., 2008). These issues may compromise the utility of neuromelanin imaging in quantifying signal alteration in the LC. Pharmacological manipulations have been used to investigate noradrenergic dysfunction in AD and PD. However, many “noradrenergic” agents are not specific to the noradrenergic system. Further, because of co-localization of synaptic NE and DA, the findings from pharmacological studies would require careful interpretation.

A few potential research directions can be explored to further our knowledge of LC function in health and illness. First, despite electrophysiological studies characterizing the roles of LC neurons in cognitive performance in behaving primates, imaging work in humans has been hampered by the small size of LC. Thus, studies combining neuromelanin imaging to more accurately localize the LC and fMRI to explore task-related activations are needed to fully understand how LC and LC connectivity with SAN, DMN, and frontoparietal network partake in task challenges. As VTA/SNc and LC are both involved in saliency response, it would be of great interest to evaluate how the two systems are differentially involved in cognitive performance and whether reward plays a specific role in differentiating noradrenergic and dopaminergic circuit functions. Likewise, studies of pharmacological manipulations can take advantage of anatomical localization of the LC and more precisely pinpoint how noradrenergic agents influence regional activity and connectivity during cognitive performance. Second, there have been neuromelanin imaging studies of the VTA/SNc in PD but less work has focused on elucidating signal changes in the LC in either PD or AD. In particular, longitudinal studies to characterize how LC signals evolve during healthy aging and in individuals with MCI or at risk of developing PD and AD would be instrumental in determining the significance of neuromelanin imaging as a tool in predicting onset and progression of the illnesses. In individuals at risk for PD, it would also be of interest to contrast findings on VTA/SNc and LC to determine how the noradrenergic and dopaminergic circuits are differentially involved in the pathogenesis of PD. Likewise, longitudinal studies of PD and AD patients undergoing treatment may benefit from knowledge whether LC neuromelanin signal intensity may serve as a biomarker of treatment outcomes. Third, the LC projects to multiple brain regions and the interaction of LC with these neural networks are likely complex and defy simple correlation analyses. More sophisticated analytical tools such as Granger causality analysis (Duann et al., 2009; Ide and Chiang-shan, 2011; Ide and Li, 2011; Hu et al., 2015) and detrended partial cross correlation (Ide et al., 2017; Ide and Li, 2018) will be useful in delineating the direction of influence and distinguish direct functional interaction from influences via a common “third party.” These analyses would be tremendously useful in confirming the hypothesis that the LC response to saliency and its projection to the SAN facilitates activity transition from the DMN to frontoparietal network for goal-directed behavior. Together, these new studies will address many unanswered questions in cognitive and clinical neuroscience, and the findings would not only advance knowledge but also better patient care.

## Author contributions

All authors listed have made a substantial, direct and intellectual contribution to the work, and approved it for publication.

### Conflict of interest statement

The authors declare that the research was conducted in the absence of any commercial or financial relationships that could be construed as a potential conflict of interest.
